# *Millepora* in Pleistocene coral reefs of Egypt

**DOI:** 10.18261/let.55.2.3

**Published:** 2022-09-07

**Authors:** Angelina Ivkić, Andreas Kroh, Abbas Mansour, Mohamed Osman, Mohamed Hassan, Martin Zuschin

**Affiliations:** University of Vienna, Department of Palaeontology, Vienna, Austria; Natural History Museum Vienna, Geological-Paleontological Department, Vienna, Austria; South Valley University, Department of Geology, Qena, Egypt; South Valley University, Department of Geology, Qena, Egypt; South Valley University, Department of Geology, Qena, Egypt; University of Vienna, Department of Palaeontology, Vienna, Austria

**Keywords:** Corals, exposure, fossil record, palaeoenvironment, taphonomy, Red Sea

## Abstract

*Millepora*, a hydrozoan coral, is a common component in modern tropical reefs throughout the world. In ecological and palaeoecological surveys, it is often grouped with scleractinian corals, which are the prevailing builders of coral reefs. On modern, current-exposed reefs in the Red Sea, *Millepora* can become the dominant coral. However, it is rarely found in fossil reefs and if present, its abundance is usually considerably lower than in modern reefs. The mismatch is often explained by a low preservation potential of the milleporid skeleton in the fossil record. We explore *Millepora* abundances in Pleistocene reefs of Egypt using 29 line transects (typically of 20 m length), and find its abundances to be comparable to that of adjacent modern reefs (between 0 and 18.8 ± 8.5% per site). Comparisons between sites with and without *Millepora* suggest that site specific environmental characteristics determine the presence of *Millepora* in the fossil reef. We conclude that a lack of preservation of habitats preferred by *Millepora* instead of the preservation potential of the hydrozoan itself is the most plausible reason for the mismatch between modern and fossil abundances.

*Millepora*, the fire coral, is a common extant hydrozoan coral on tropical reefs throughout the world. The genus is distinct due to its gastrozooids emerging from the gastropores, which are surrounded by several dactylozooids protruding from dactylopores (Boschma 1951). Identification to species level is difficult and in addition to growth form requires a detailed analysis of skeletal features, such as the pores (e.g. amount, size) (Arrigoni *et al*. 2018; Boissin *et al*. 2020). Today, *Millepora* is present in tropical and subtropical reefs in the Atlantic and Indo-Pacific at depths with sufficient light levels (see Dubé *et al*. 2019 for distribution maps). While it is a keystone taxon in many recent reefs and can be locally abundant, it usually covers less than 10 percent of the substratum over a whole reef (Lewis 1989; Lewis 2006). However, its distribution depends on physical factors, such as water depth and exposure to water energy (Montaggioni & Braithwaite 2009). In some instances, *Millepora* is one of the dominant corals in a reef zone and, therefore, in the Caribbean (Geister 1977; Graus & Macintyre 1989) and the Red Sea (Loya 1972) reef zones have been named after the fire coral. *Millepora* is found in environments with different physical characteristics (e.g. Dubé *et al*. 2017; Lewis 1989), but if highly abundant, it is always in exposed and often in shallow settings (Dubé *et al*. 2017; Rioja-Nieto & Álvarez-Filip 2019). The exposure can be either to waves (Dubé *et al*. 2017; Geister 1977) or currents (Riegl & Piller 1997; Riegl & Velimirov 1994). The Red Sea is particularly well known for its high *Millepora* abundances (e.g. Mergner & Schuhmacher 1974; Sheppard & Sheppard 1991), with *Millepora dichotoma* Forsskål, 1775 (Fig. 1) covering up to 36% of the substratum in reefs off Eilat, Israel (Loya & Slobodkin 1985). On current exposed sites of the Red Sea, in both off- and onshore reefs and down to 20 meter depth, *Millepora* is often described as the dominant coral (Riegl & Piller 1997; Riegl & Velimirov 1994; Riegl *et al*. 2012).

While *Millepora* is a common component in modern reefs throughout the world, it is rarely recorded in the fossil record. One of the earliest records of *Millepora* is from the Cretaceous of Northern Spain (Rehfeld & Ernst 1998), followed by records from the middle Eocene of the Caribbean (Stemann 2003). Compared to Scleractinia, the fossil record of *Millepora* is scarce. Only during the Pliocene and Pleistocene do records of the fire coral increase (e.g. Baarli *et al*. 2013; Budd *et al*. 1999; El-Sorogy 2008; Greenstein *et al*. 1998; Petuch 1986). Quantitative studies in the Last Interglacial (~125 ka ago) emphasize the mismatch between fossil and extant occurrences of milleporids (e.g. Greenstein *et al*. 1998) and it is striking that even in the youngest fossil record *Millepora* is less abundant than in modern tropical reefs.

One possible explanation for this mismatch could be a lower preservation potential of the milleporid skeleton compared to the scleractinian skeleton (Greenstein *et al*. 1998; Hunter & Jones 1996). Both are Cnidaria, which have two cell layers with a mesoglea in between and often live in symbiosis with zooxanthellae in their entoderm and build a skeleton of aragonite. Overall both, the morphological and the ecological similarities of Scleractinia and *Millepora* are striking (Lewis 2006). However, stony corals build corallites, while fire corals have gastropores and dactylopores, which accommodate two different kinds of polyps. Furthermore, there are differences in the microstructure of the aragonite fibres: the milleporid skeleton is built of short fibres with poor crystallization while in scleractinian corals long bundles of fibres are common (Cuif *et al*. 2010).

Another possible explanation for the low amount of *Millepora* in the fossil record is related to its habitat preference. In modern coral reefs it is usually described as being abundant only in exposed (to currents or waves) and often shallow environments. The *Millepora* zone in the Caribbean is on high energy reef crests (Geister 1977), in the Red Sea on exposed reef crests and upper reef slopes (Loya 1972; Riegl & Piller 2000; Riegl & Velimirov 1994). For the Indo-Pacific, it is described as part of the ‘robust-branching coral assemblage’, which is associated with the exposed reef crest and upper reef slope (Montaggioni 2005). Therefore, under the assumption of niche conservatism, *Millepora* would only be abundant in palaeoenvironments classified as either current- or wind- and wave-exposed and placed along the palaeo-reef crest and upper reef slope. However, this also implies that those habitats need to be preserved in the fossil record.

Given the importance of *Millepora* in modern reefs and therefore its influence on the interpretation of comparisons between recent and fossil coral reefs, it is essential to understand the reasons of a potential mismatch between fossil and recent presences of the fire coral. In this paper, we examine the mismatch quantitatively by documenting *Millepora* abundance in the Pleistocene reefs of Egypt and comparing it to extant Red Sea coral reefs.

## Geological setting

Fossil fringing reef terraces can be found along most of the Egyptian coast and are only interrupted by erosional valleys and alluvial fans (Mansour & Madkour 2015; Plaziat *et al*. 2008). Their formation was controlled by the cyclicity of Pleistocene ice ages, with high sea levels during interglacials resulting in fringing reefs along the coast (Plaziat *et al*. 2008). Older Pleistocene reef units were tectonically raised up to 50 metres, while the Eemian terraces are usually around 4 metres above current sea level (Plaziat *et al*. 1998). It is under discussion whether they have been tectonically uplifted as well (Lambeck *et al*. 2011), or whether they are close to their original altitude with lowering of eustatic sea level as the only reason for their exposure on land (Plaziat *et al*. 2008).

The reef unit closest to the modern coastline can be of Holocene origin, but it is usually not more than 1 metre above current sea level (El-Asmar 1997). In most cases, however, the fossil reefs adjacent to the modern coast can be assigned to MIS5e (Eemian, Last Interglacial, Late Pleistocene), with Middle Pleistocene reefs below the Eemian reefs or further inland (Casazza 2017; Plaziat *et al*. 2008). The fossil reefs investigated in this study lie either immediately adjacent or only a few metres away from the current coast line. Their height above current sea level is between 0.5 and 10 metres (Table 1).

**Fig 2:**
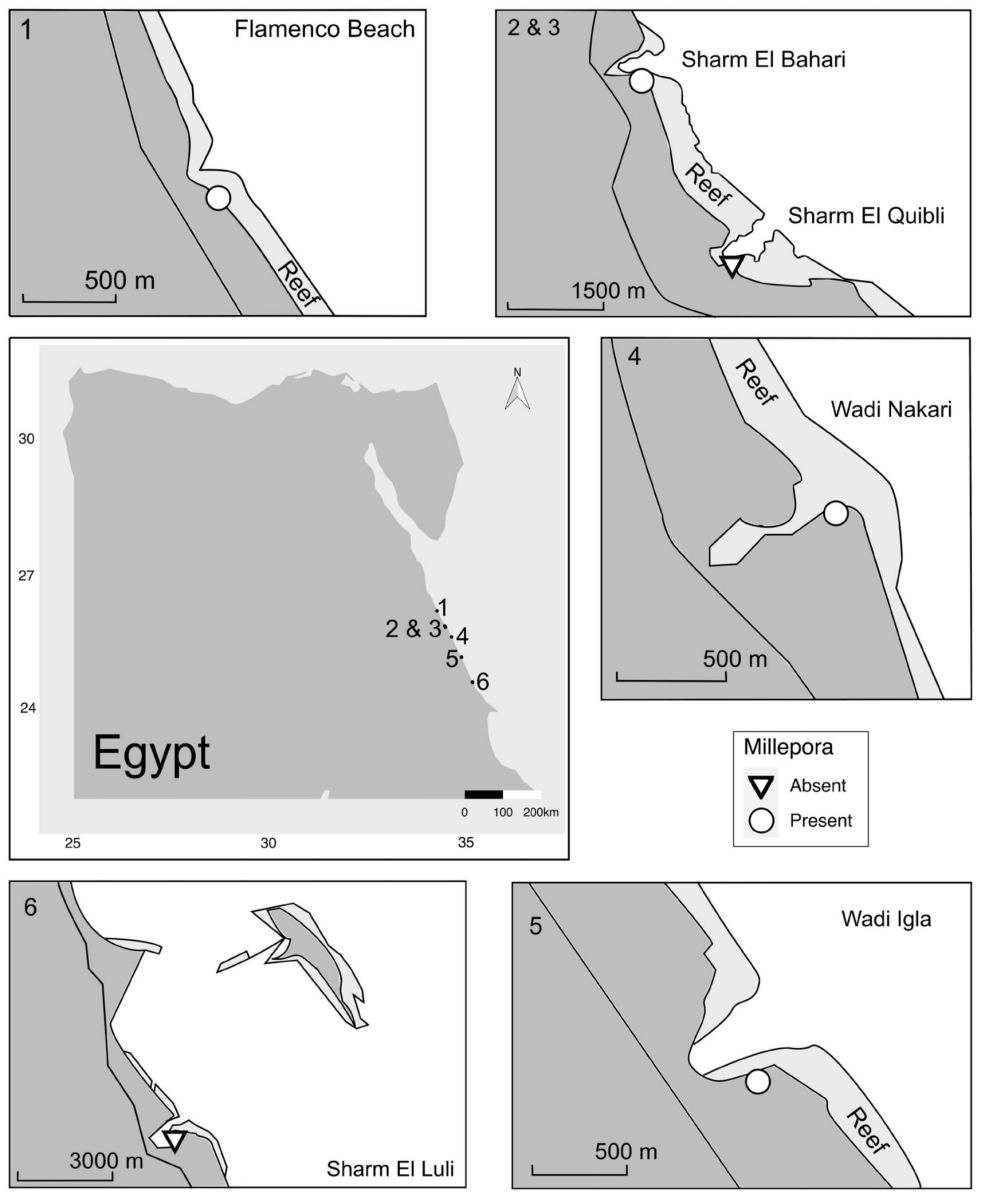
Studied fossil transect localities along the Egyptian Red Sea coast. 1 = Flamenco Beach, 2 = Sharm El Bahari, 3 = Sharm El Quibli, 4 = Wadi Nakari, 5 = Wadi Igla, 6 = Sharm El Luli.

The ages of all our fossil reef localities except for Flamenco Beach (Fig. 2) were extensively reviewed and discussed by Plaziat *et al*. (2008). Based on the geology of the region, we were able to identify all of the reefs in this study with the exception of Wadi Igla as previously dated MIS5e reefs. The investigated reef in Wadi Igla was some metres further inland and had a higher elevation and therefore, it is a pre-MIS5 reef (older than 130 ky).

The fossil reefs consist of coral framestones, coral bafflestones, rudstones dominated by corallinacean algae (partially with rhodoliths and molluscs), and packstone, filling the gaps between the coral framestone.

## Material and methods

The investigated fossil reefs were all located on the Egyptian coast of the Red Sea between 26.2°N and 24.6°N (Fig. 2). The R package ‘ggmap’ was used for visualization (Kahle & Wickham 2013). Six sites were considered for this study (ordered from North to South): (1) Flamenco Beach; (2) Sharm El Bahari; (3) Sharm El Quibli; (4) Wadi Nakari; (5) Wadi Igla; and (6) Sharm El Luli. At each reef locality at least three transects were sampled. In Sharm El Luli, Sharm El Bahari, and Wadi Nakari the outcrops allowed us to sample from two reef zones: shallower sites close to the palaeo-reef edge, consecutively called ‘reef edge’, and deeper sites, which corresponded to the shallow reef slope and are consecutively called ‘reef slope’ Due to outcrop conditions, the transects in Sharm El Quibli represented only reef edge, while those at Flamenco Beach only reef slope habitats. In Wadi Igla, five transects were sampled between 5 and 10 metres above present-day sea level and grouped as ‘pre-MIS5e’ reefs without depth differentiation.

In some reef localities (Sharm El Bahari reef edge & slope, Flamenco Beach reef slope, Wadi Nakari reef edge & slope and Sharm El Luli reef edge) the encrusting gastropod *Ceraesignum* cf. *maximum* (G.B. Sowerby I, 1825), which is an indicator for shallow reef habitats (Zuschin *et al*. 2001), was present.

The localities were sampled during two field trips in February/March and November 2019. The data were collected using Line Intercept Transects (LIT): a tape measure was laid out at a random starting point following the depth contour. We aimed for a length of 20 metres per transect (English *et al*. 1997) but due to inaccessibility or poor exposure some transects were shorter (LT12 was the shortest with 14.12 metres). The substrate underlying the tape was identified to an accuracy of one cm. Where possible, corals were identified to genus level in the field. If this was not possible, specimens were photographed and sampled for detailed identification in the laboratory. Furthermore, we differentiated between rudstone, packstone, bafflestone, and framestone using the extended Dunham carbonate classification (Dunham 1962; Embry & Klovan 1971). Corals intersecting the tape that were not in growth position were counted as tilted corals. 2099 datapoints were collected in total. The complete data table is available at Dryad doi:10.5061/dryad. pzgmsbcpw. For corals that could not be identified to genus level, datapoints were removed prior to analysis (1.7% of datapoints). Furthermore, for some corals of the family *Pocilloporidae* we were not able to distinguish between the genera *Pocillopora* and *Stylophora* due to poor preservation. Every occurrence of *Pocilloporidae* indet was therefore proportionally split according to their overall abundances in all transects (91% *Pocillopora*, 9% *Stylophora*). Molluscs, coralline algae, and tilted corals were included in the category ‘sediment’ for the purpose of this study. The percent abundance of the categories sediment, *Millepora*, and other corals (Scleractinia and *Sinularia* holdfast) were calculated for each transect (Table 1). Transects were grouped by localities and ages, and for MIS5e reefs, reef edge and reef slope within each locality are indicated. Mean percentages of *Millepora*, the sediment, and the eight most abundant scleractinian genera (collectively called categories) were calculated for each transect (Table 2). Additionally, after grouping the transects by *Millepora* presence/absence, the mean percent abundances of the most abundant categories and their 95% binomial confidence intervals were calculated for the two groups using the arcsin square root transformed data and differences within individual categories were tested using a Wilxoc test.

Statistical analyses were performed with proportional relative abundances for each transect, which were square root transformed to deemphasize the importance of the most dominant taxa. Data on *Millepora* were excluded a priori to avoid circular reasoning and nonmetric multidimensional scaling (NMDS) based on the Bray-Curtis dissimilarity was used to visualize differences between the predefined groups (*Millepora* presence/absence, localities, ages and reef slope or reef edge for MIS5e reefs). To test for differences between groups we used permutational multivariate analysis of variance (PERMANOVA) (Anderson 2001). Furthermore, similarity percentages (Simper) were calculated to find the species contributing most to the dissimilarity between reefs with and without *Millepora* (Clarke 1993). All statistical analyses were performed in R Version 3.5.2 (R Core Team 2018) using the ‘vegan’ package (Oksanen *et al*. 2019).

## Results

*Millepora* sp. was found in 20 of 29 transects (~69%) (Table 1). The sites with *Millepora* presence are: Wadi Igla, Sharm el Bahari, Flamenco Beach, and Wadi Nakari. In the reef transects in Sharm el Luli and Sharm el Quibli, no *Millepora* was found.

The transect with the highest abundance of *Millepora* (65.1%) is LT02, a pre-MIS5e reef in Wadi Igla, followed by a much lower *Millepora* abundance (19.4%) in LT01 at the same locality and 19.4% in LT75 (Wadi Nakari, MIS5e) (Table 1). The transect with the lowest abundance of *Millepora* is LT78 with 0.3%, a reef slope transect at Wadi Nakari (MIS5e).

Transects grouped by locality, age and palaeodepth show that the pre-MIS5e reef transects in Wadi Igla had the highest abundance of *Millepora*, followed by the MIS5e reef edge in Sharm El Bahari (Table 2). *Millepora* abundances ranged between 18.7 ± 8.5% in the pre-MIS5e reef of Wadi Igla and 0.9 ± 0.4% at the MIS5e reef slope in Wadi Nakari. *Porites* was the scler-actinian with the highest abundance in all reefs except for the MIS5e reef slope in Sharm El Bahari. The mean abundances of *Porites* were highest in the reefs with no *Millepora* (Sharm El Luli and Sharm El Quibli).

**Fig 3:**
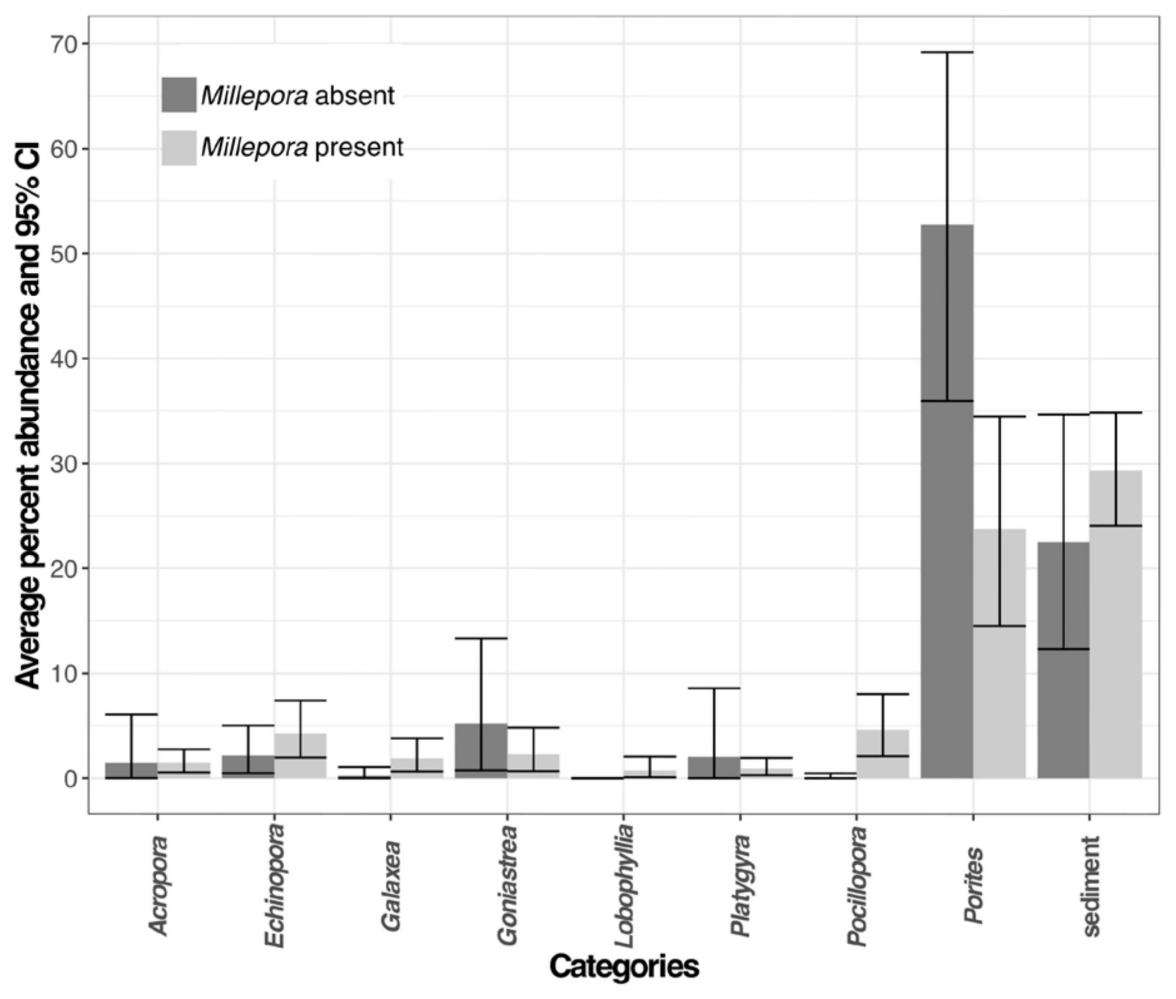
Average percent abundances of the ten most abundant categories grouped by *Millepora* presence and their 95% confidence intervals.

Sediment and *Porites* are the most abundant categories in reefs with and without *Millepora* (Fig. 3). However, the relative abundances of categories differ between the two groups. *Porites* is more abundant in reefs that do not contain any *Millepora* (Wilcox test, p = 0.0095), while reefs that contain *Millepora* have higher percentages of *Pocillopora* (Wilcox test, p = 0.0004) and *Lobophyllia* (Wilcox test, p = 0.0312) (Fig. 3).

**Fig 4:**
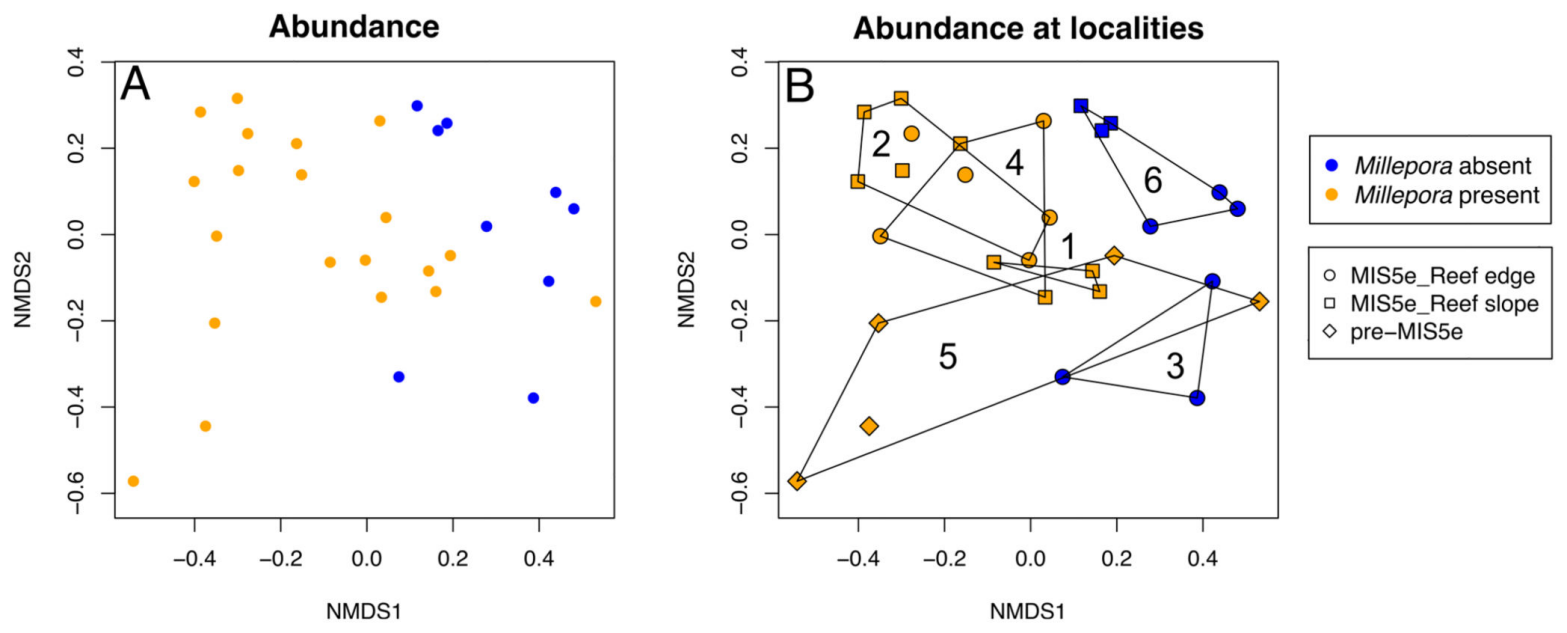
NMDS analysis of all fossil transects using square root transformed abundance data with *Millepora* abundances excluded a priori. Hulls indicate localities: 1 = Flamenco Beach; 2 = Sharm El Bahari; 3 = Sharm El Quibli; 4 = Wadi Nakari; 5 = Wadi Igla; and 6 = Sharm El Luli. Stress is 0.17. A indicates *Millepora* absence and presence. B additionally indicates localities, ages and reef edge and reef slope for MIS5e reefs.

The PERMANOVA (with *Millepora* abundances excluded a priori) showed a clear distinction between reefs with and without *Millepora* and explained 14.3% of the variation in the dataset (R^2^~0.143, p = 0.002) (Fig. 4A). For the same dataset, the differences between localities and ages were significant and explained 43.5% (R^2^ ~0.435, p = 0.001) and 9.6% (R^2^~0.096, p = 0.01) of the variation, respectively. Reef edge and reef slope of the MIS5e reefs also differed significantly (p = 0.035, R^2^~0.10) (Fig. 4B). Simper results indicated that the categories *Porites*, *Pocillopora*, sediment, *Goniastrea*, and *Echinopora* accounted for 46.2% of the differences between reefs with and without *Millepora*.

**Fig 5:**
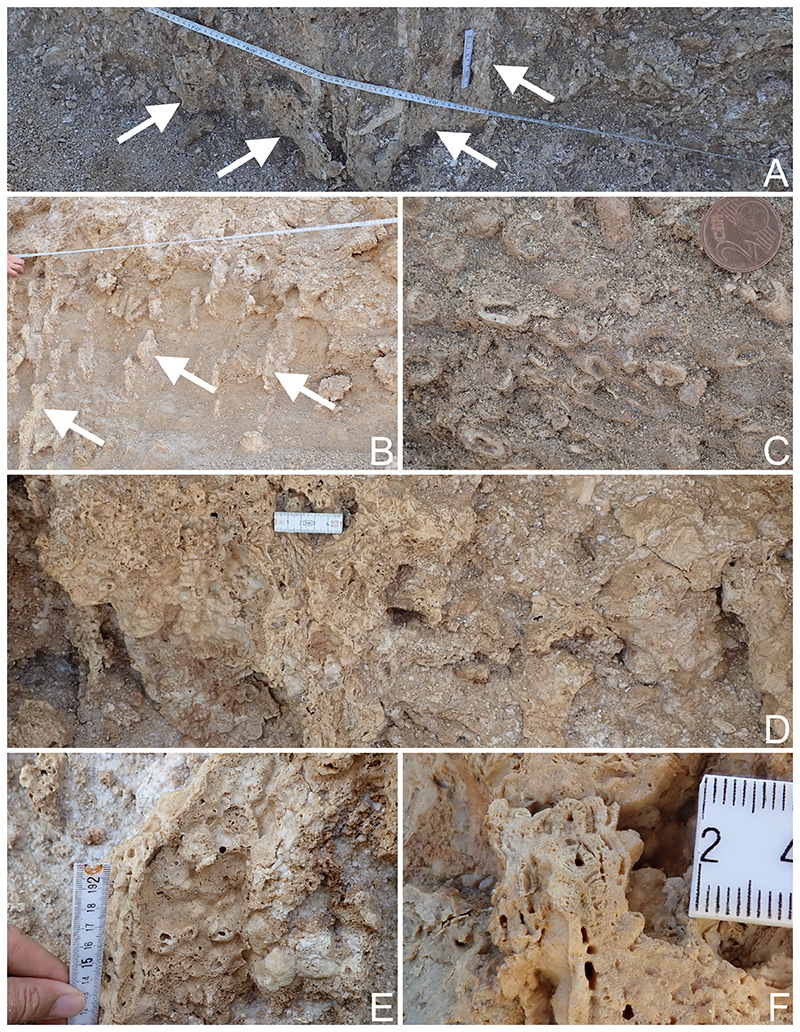
*Millepora* examples in the fossil terraces of Egypt. A, B, fossil platy *Millepora* sp. from Wadi Igla and Wadi Nakari; C, cross-cut through fossil fan shaped *Millepora* sp. from Wadi Nakari; D, fossil *Millepora* sp. from Flamenco Beach; E–F, close-ups of fossil *Millepora* sp. from Flamenco Beach and Sharm El Bahari. Coin for scale has a diameter of 18.75 mm (C).

The most common appearance of *Millepora* in the fossil reef is with a blade-shaped growth form, as is typical for *Millepora platyphylla* Hemprich & Ehrenberg, 1834 (Fig. 5A-B, D-F). The fan-shaped growth form, common in *Millepora dichotoma* Forsskål, 1775, was identified as well (Fig. 5C). Some encrusting growth forms strongly resembled *Millepora exaesa* Forsskål, 1775 in the field, but pore characters were not well enough preserved to exclude other possibilities.

## Discussion

### *Occurrence of* Millepora *in the fossil record*

Fossil corals that were assigned to the milleporids in the past were often not compared to recent milleporids, which led to many wrong identifications (Boschma 1951). The first record that appears in the Paleobiology Database (PBDB) using the term *Millepora* is from the late Jurassic of Croatia (Milan 1969), but the species is now considered to be a coralline red algae (Basso *et al*. 2004). The earliest putative record of *Millepora* is from the Cretaceous of Northern Spain (Rehfeld & Ernst 1998). Later records are from the Eocene and Upper Oligocene of the Caribbean (Stemann 2003). Altuna *et al*. (2007) describe a new species from the Eocene of Spain. However, this record is doubtful because the figured specimen shows only one kind of pores. Furthermore, they and Darga (1992) list additional species, which were not confirmed as milleporids by Boschma (1951). In the Pliocene and Pleistocene, the number of *Millepora* findings in the fossil record increases, with reports from the Caribbean (e.g. Budd *et al*. 1999; Gischler 2007; Greenstein *et al*. 1998; Hattin & Warren 1989; Petuch 1986), Santiago Island (Baarli *et al*. 2013), and the Red Sea (Bosworth *et al*. 2018; El-Sorogy 2008; Kahal *et al*. 2020).

It is surprising how rare *Millepora* is in the fossil record compared to how common it is in modern reefs, where it is often described as a keystone species, which covers around 10% of the substratum and can be locally dominant (Lewis 1989; Lewis 2006). Montaggioni & Braithwaite (2009) emphasize the mismatch for the Caribbean, where many Pleistocene reefs are present but comparatively few fire corals were recorded. After a comparison of life, death, and fossil assemblages in reefs of the Bahamas, Greenstein *et al*. (1998) found that *Millepora* is more common in the life assemblage than it is in the death and fossil assemblage. Even at the Red Sea, where it is a dominant coral on modern current-exposed reefs and where many Pleistocene reefs are present, *Millepora* has been described as scarce in the Pleistocene fossil record (Mewis 2006). Alexandroff *et al*. (2016) describe it as one of the most abundant genera on modern Egyptian reefs, especially on the shallow reef slope, while they report covers of less than 1 percent in the investigated fossil reefs close-by. Casazza (2017), who studied Pleistocene reefs of Egypt, did not report any *Millepora*.

Our abundances of *Millepora* from Pleistocene reefs in Egypt are comparable to modern abundances of this hydrozoan. Under certain physical conditions, *Millepora* can become dominant in modern reefs. Riegl & Piller (1997) state that reefs in northern Egypt (Bay of Safaga), if current-exposed, can be ‘more or less entirely dominated by *M. dichotoma’*. The pre-MIS5e reef in Wadi Igla had high abundances of *Millepora* with 18.7 ± 8.5%. The reefs in Sharm El Bahari, Flamenco Beach, and Wadi Nakari had mean abundances of *Millepora* between 11.7 ± 0.1% and 0.9 ± 0.4%, while we found no *Millepora* in Sharm El Luli and Sharm El Quibli. The percentage of fire coral of the total intercepts for modern Egyptian reefs usually ranges between 0 and 25% (Riegl & Velimirov 1994), depending on the exposure to waves and currents. At Sharm El Bahari, we can directly compare the *Millepora* abundance between modern and Late Pleistocene reefs. There, *M. dichotoma* and *M. platyphylla* made up about 5% of the transects in the modern reef (Dar *et al*. 2012) and 4.9% in the fossil reef investigated in our study. Overall our abundance data on *Millepora* from Pleistocene coral reefs in Egypt compares well with the numbers on modern reefs, especially from the Red Sea. Therefore, at first sight, our results do not indicate a bias in preservation potential under the assumption that the community composition was similar between the Pleistocene and today.

### Palaeoenvironmental setting as a key factor

We were able to differentiate between two depths in most of the investigated fossil reefs: shallower sites closer to the reef edge and deeper sites along the shallow reef slope. Fossil reefs accurately preserve the depth zonation (Edinger *et al*. 2001) and in the MIS5e reefs of Egypt the zonation was captured in the morphology (reef flat, reef edge, reef slope).

The site with the highest abundance of *Millepora* was the pre-MIS5e reef in Wadi Igla with 18.7 ± 8.5%. The Scleractinia with the highest abundance in this reef were *Porites* with 18.7 ± 14.1%, followed by *Pocillopora* with 4.9 ± 7.5% and *Acropora* with 1.8 ± 2.5%. One of the transects in this reef, LT02, had an exceptionally high abundance of *Millepora* with 65.1%. The combination of high abundances of *Millepora*, *Pocillopora, Porites* in more sheltered and *Acropora* in more exposed areas, and high coral cover (percentage of sediment: 34.5 ± 2.6%) is characteristic of a so-called *‘Millepora* reef’ described by Riegl & Piller (1997). The *Millepora* reef is defined by the high abundances of the fire coral and is typical for current-exposed reefs in the Red Sea.

Sharm el Bahari, Flamenco Beach, and Wadi Nakari (all MIS5e) had lower percentages of milleporids than Wadi Igla, but all of the localities had high abundances of *Porites* (Table 2), which would correspond to a semi-exposed to sheltered reef.

We found no *Millepora* in Sharm El Luli and Sharm El Quibli, which were both dominated by *Porites*. The two sites are today in protected bays (see Fig. 2) and not exposed to currents or waves. Assuming similar conditions during the Pleistocene and based on the coral composition at the sites, the reefs would correspond to the modern ‘leeward *Porites* assemblage’ of Riegl & Piller (1997). They describe such assemblages as being dominated by *Porites* and sometimes forming a ridge, completely composed of *Porites*. Further down, along the reef slope, tabular *Acropora* become frequent (Riegl & Piller 1997; Riegl & Velimirov 1994). Sharm El Quibli is the only locality of all our investigated fossil reefs, where we found tabular *Acropora*.

The abundances of *Millepora* and the modern exposure of the investigated sites suggest palaeoexposure to be a decisive factor for *Millepora* presence. As it is difficult to reconstruct exposure of fossil reefs, we investigated the community composition of the localities by summarizing all sites with *Millepora* presence and all sites with *Millepora* absence to test this hypothesis. The sites that contain milleporids have a lower mean *Porites* abundance and higher mean *Pocillopora* abundance. Due to poor preservation, we were not able to identify *Pocillopora* to species level in the fossil record. However, the genus is known to be abundant in shallow waters and *P. verrucosa* is very common on modern reef slopes with moderate exposure (Sheppard & Sheppard 1991).

Furthermore, we investigated the palaeo-habitat in some fossil reefs in which we could confirm *Millepora* records with certainty. Budd *et al*. (1999) reconstructed the habitats with *Millepora* presence as having moderate exposure and shallow to intermediate water depth in the Buenos Aires trend (Pliocene) and as exposed, high energy reefs with shallow water depth in the Empalme trend (Pleistocene) in Costa Rica. Petuch (1986) was able to record branching *Millepora alcicornis* from a former high-energy reef crest from the Pliocene of Southeastern Florida, and Baarli *et al*. (2013) found the same species in a shallow high energy setting in the Pleistocene of Santiago Island. At the Red Sea, El-Sorogy (2008) found *Millepora dichotoma* together with *Pocillopora verrucosa* in reef crest facies from the Pleistocene. All of those records suggest a similar palaeoenvironment: a shallow, highly to moderately exposed reef crest to reef slope. Gischler (2007) studied Pleistocene reefs underneath recent coral reefs of Belize by coring and was able to distinguish different reef facies. In the reef facies, which was dominated by *Acropora palmata* and which he reconstructed as the reef crest, he found *Millepora*. Interestingly, he found *Millepora* even though the limestone was diagenetically altered.

Based on all the above-mentioned studies and our own study of Pleistocene reefs in Egypt, we suggest that the palaeoenvironmental setting is decisive for the presence or absence of *Millepora* in the fossil record. Site specific differences explain a high percentage of the variability of our data (43.5%) and exposure is one key parameter distinguishing modern Red Sea reef localities (e.g. Riegl & Piller 1997; Sheppard & Sheppard 1991).

It is difficult to reconstruct palaeoenvironmental settings from fossil reefs, especially the exposure. However, to deduce the presence or absence of the hydrozoan genus in the past, it is essential to investigate habitats, which fulfill the niche requirements of the genus. *Millepora* is a common species on modern reefs, but usually with relatively low overall reef cover and only locally high abundances, depending on the environment (Lewis 2006). This has to be considered when comparing fossil with modern reef transects. For example, Greenstein *et al*. (1998) argued that the difference in species richness from live to dead to fossil assemblage partially results from the absence of two of the three *Millepora* species present in the modern reefs (one is absent in the dead and one is absent in the fossil assemblage) and concluded a higher susceptibility of the hydrozoan skeleton to diagenesis. However, they also mentioned another possibility for the mismatch between the modern and the Pleistocene assemblage: differences in the environmental setting between the modern and the fossil reef. The habitat they investigated in the modern reef is a mid-shelf patch reef, while the fossil reef habitat represents a protected back reef and reef tract. Based on this information and the modern preference of exposed habitats by the fire coral, we would expect the milleporids in the fossil site investigated by Greenstein *et al*. (1998) to be less abundant than in the modern reef because of a difference in the (palaeo) environments studied.

Further decisive factors for the presence of *Millepora* could be the ecological succession and frequency of disturbance on a reef, as in the Red Sea it has been described as an opportunistic species and rapid colonizer after disturbances (Loya 1976). Additionally, the water depth, suspension load and topography (Riegl & Piller 1997), which control light availability, have to be considered. It is difficult to reconstruct any of those environmental factors in fossil reefs, but the difference between localities and, for the MIS5e reefs, depth, even after excluding *Millepora* from our data, is significant and explains a substantial amount of the variation in our data. This shows that the site specific characteristics need to be considered when attempting to make meaningful comparisons between fossil and recent reefs.

### Millepora *presence as a proxy for preservation?*

Another reason for the scarcity of milleporids in the fossil record could be a lower preservation potential of hydrozoan taxa compared to the Scleractinia (Greenstein *et al*. 1998; Hunter & Jones 1996). Susceptibility to diagenesis and taphonomic reworking are major factors that have to be considered when interpreting the fossil record. Fossil reefs are primarily made of CaCO_3_, which is prone to dissolution and recrystallisation, especially in meteoric environments (Constantz 1986). *Millepora* and Scleractinia are both made up by aragonite, whose susceptibility to dissolution mainly depends on the amount of reactive surface area, which is dependent on the aragonite fibre width and the intragranular porosity (Constantz 1986). *Millepora* has shorter fibres than Scleractinia (Cuif *et al*. 2010) and therefore, a higher susceptibility of the hydrozoan compared to scleractinian corals is possible.

It is noteworthy that the Pleistocene *Millepora* were dominantly found with platy growth form, while on modern Red Sea reefs, *M. dichotoma* (fan-shaped growth form) is usually described as the most common milleporid (Loya 1972; Riegl & Piller 1997; Riegl & Velimirov 1994). This could either be explained by a change in community composition from predominantly platy to branching growth form since the Pleistocene or alternatively it could result from a difference in preservation potential between the two growth forms.

An additional explanation for the high abundances of *Millepora* in our study site could be an exceptionally good preservation of the studied reefs in Egypt. We generally expect highest preservation potential in fossil reefs of young geological age and under arid environmental conditions. As our fossil reefs are from the Pleistocene, they are of geologically young origin and today the Egyptian coast is part of a hot, hyperarid climate with very little rainfall. Growth of speleothems during MIS5.5 in Egypt (El-Shenawy *et al*. 2018) and global climate models (Jennings *et al*. 2015) suggest more precipitation along the Egyptian coast during MIS5e. However, the absence of speleothem growth (El-Shenawy *et al*. 2018) and the models used in Jennings *et al*. (2015) suggest an arid climate in Egypt for the investigated periods after MIS5e (115 ka, 104 ka, 56 ka and 21 ka).

This would imply that our investigated fossil reefs faced limited exposure to aggressive meteoric waters, which probably enhanced preservation potential of: (1) the fire coral itself; and (2) of habitats, which are preferred by *Millepora*.

Inversely, the presence of *Millepora* in fossil reefs could serve as a proxy for exceptionally good preservation of the reefs (consider that the reverse is not valid as *Millepora* is abundant only in specific habitats). However, as described earlier in detail, fossil *Millepora* was found in other parts of the world with a higher precipitation rate such as Costa Rica (Budd *et al*. 1999), Southeastern Florida (Petuch 1986), or Cape Verde (Baarli *et al*. 2013).

Furthermore, we found the highest abundance of *Millepora* in Wadi Igla, which is older than MIS5e. As early MIS9, MIS7.3 and MIS5.5 were all periods of increased precipitation in Egypt (El-Shenawy *et al*. 2018), the reefs in Wadi Igla must have been exposed to aggressive meteoric waters and nevertheless, the fire coral is well preserved in those fossil reefs.

Therefore, we conclude that an arid climate can facilitate the preservation of *Millepora* in fossil reefs, but that it can be found in fossil reefs even under the influence of meteoric waters if the appropriate palaeoenvironments are preserved and investigated. An important implication for all studies correlating modern with fossil coral reefs is that the environments (e.g. exposure and depth) in both time periods must match for meaningful comparisons.

## Conclusions

We found no major difference between *Millepora* abundances of Pleistocene and modern Red Sea reefs and conclude that the fire coral does not have an exceptionally low preservation potential in fossil reefs. Rather, the preservation of palaeoenvironments, which reflect the niche of the milleporid, is necessary to find fire corals in the fossil record. The physical factor most decisive for *Millepora* presence is exposure to water energy, combined with a depth with sufficient light availability. Other factors increasing the preservation potential at our fossil sites might be the young geological ages and a geographical location with an arid climate, which is advantageous to preservation. High initial abundances of *Millepora*, as they are known for the modern Red Sea, may further mitigate a lower preservation potential of the hydrozoan skeleton.

## Figures and Tables

**Fig 1:**
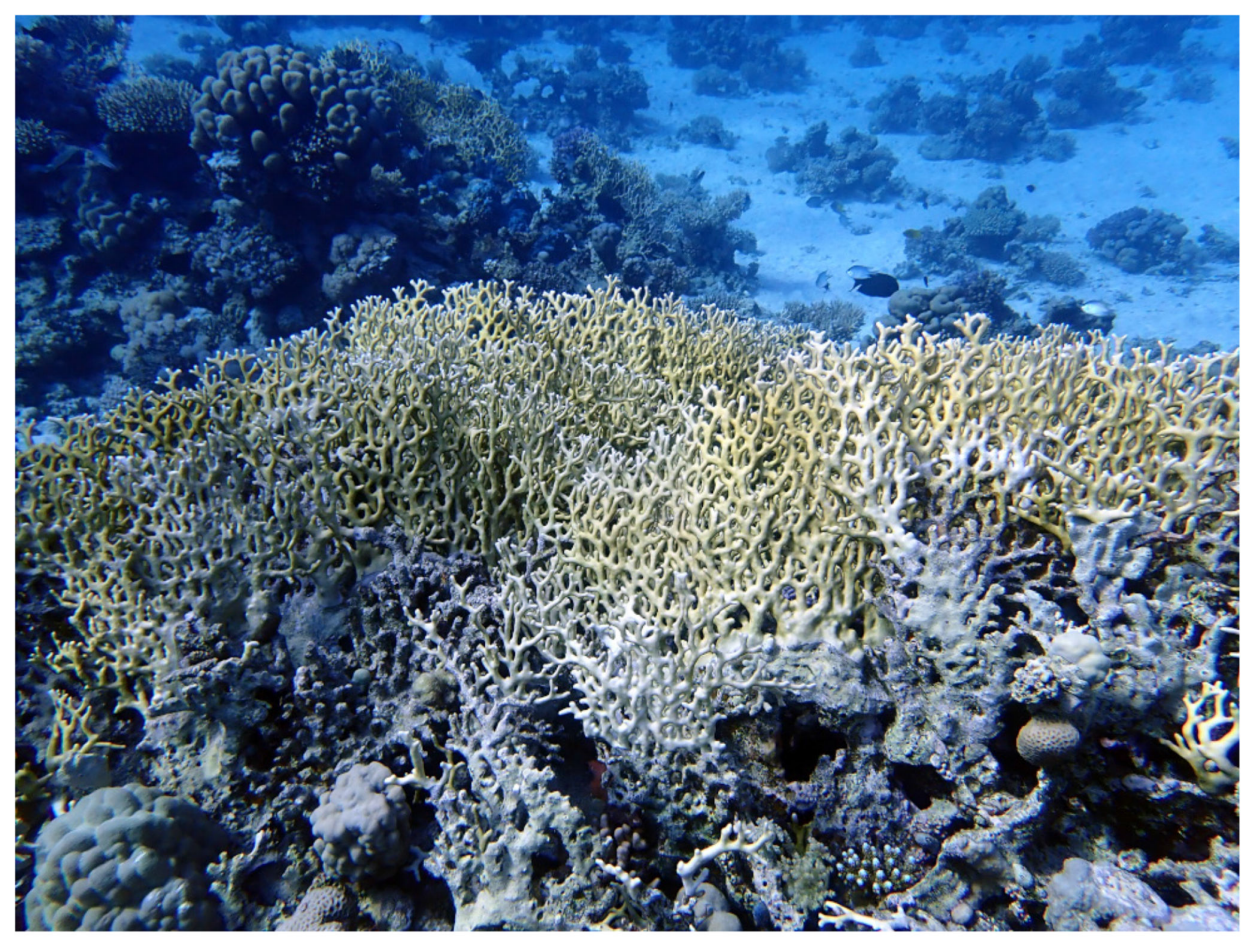
*Millepora dichotoma* from a modern Egyptian reef. Photo: Angelina Ivkić.

**Table 1:** Summary of all fossil transects and their characteristics

Transect	Locality	Height above current sea level [m]	Length of transect [m]	Age	Depth category	Percentage of other corals (%)	Percentage of *Millepora* (%)	Percentage of sediment (%)
**LT01**	Wadi Igla	10.0	19.6	pre-MIS5e	–	15.9	19.4	64.8
**LT02**	Wadi Igla	9.0	19.5	pre-MIS5e	–	8.1	65.1	26.7
**LT03**	Wadi Igla	5.0	17.2	pre-MIS5e	–	69.0	9.0	22.0
**LT05**	Wadi Igla	7.0–8.0	16.6	pre-MIS5e	–	51.3	13.9	34.8
**LT06**	Wadi Igla	9.0	19.6	pre-MIS5e	–	66.3	2.5	31.3
**LT60**	Sharm El Bahari	1.0	21.5	MIS5e	Reef slope	66.5	0.7	32.8
**LT61**	Sharm El Bahari	1.0	21.6	MIS5e	Reef slope	79.1	1.1	19.9
**LT62**	Sharm El Bahari	1.0	23.7	MIS5e	Reef slope	57.6	3.7	38.7
**LT63**	Sharm El Bahari	1.0	20.0	MIS5e	Reef slope	71.4	1.6	27.1
**LT68**	Sharm El Bahari	3.5	22.4	MIS5e	Reef edge	75.3	12.3	12.4
**LT69**	Sharm El Bahari	3.0	19.3	MIS5e	Reef edge	69.3	13.2	17.5
**LT70**	Sharm El Bahari	3.0–4.0	20.6	MIS5e	Reef edge	78.0	9.8	12.3
**LT72**	Flamenco Beach	1.2	20.2	MIS5e	Reef slope	65.0	0.6	34.5
**LT73**	Flamenco Beach	1.5-2.5	19.2	MIS5e	Reef slope	55.3	10.5	34.2
**LT74**	Flamenco Beach	1.5	23.7	MIS5e	Reef slope	61.7	18.3	20.0
**LT75**	Wadi Nakari	4.0	19.4	MIS5e	Reef edge	48.8	19.4	31.8
**LT76**	Wadi Nakari	4.0–5.0	20.6	MIS5e	Reef edge	73.1	1.7	25.3
**LT77**	Wadi Nakari	2.0	21.3	MIS5e	Reef edge	56.0	6.2	37.8
**LT78**	Wadi Nakari	0.5	18.9	MIS5e	Reef slope	55.5	0.3	44.2
**LT79**	Wadi Nakari	0.5–1.0	20.9	MIS5e	Reef slope	68.0	1.9	30.1
**LT07**	Sharm El Luli	3.0	19.9	MIS5e	Reef edge	78.3	0.0	21.7
**LT09**	Sharm El Luli	4.0–5.0	23.2	MIS5e	Reef edge	87.9	0.0	12.1
**LT11**	Sharm El Luli	1.5	20.5	MIS5e	Reef slope	66.5	0.0	33.5
**LT12**	Sharm El Luli	1.2	14.1	MIS5e	Reef slope	48.6	0.0	51.4
**LT13**	Sharm El Luli	1.5	16.6	MIS5e	Reef slope	71.3	0.0	28.7
**LT14**	Sharm El Luli	4.0	26.2	MIS5e	Reef edge	95.7	0.0	4.3
**LT64**	Sharm El Quibli	3.0	21.6	MIS5e	Reef edge	72.5	0.0	27.5
**LT65**	Sharm El Quibli	3.0	12.4	MIS5e	Reef edge	77.0	0.0	23.0
**LT66**	Sharm El Quibli	3.5–4.0	22.1	MIS5e	Reef edge	90.0	0.0	10.0

**Table 2:** Transects grouped by localities and palaeo-depths, sorted by *Millepora* abundance (decreasing)

Locality	Age	Depth category	Number of replicates	% *Millepora* ± standard deviation	% Sediment ± standard deviation	Scleractinia highest abundance	% Scleractinia highest abundance ± standard deviation
**Wadi Igla**	pre-MIS5e	–	5	18.7 ± 8.5	34.5 ± 2.6	*Porites*	18.7 ± 14.1
**Sharm El Bahari**	MIS5e	Reef edge	3	11.7 ± 0.1	14.8 ± 0.4	*Porites*	35.4 ± 6.0
**Sharm El Bahari**	MIS5e	Reef slope	4	1.6 ± 0.2	29.4 ± 0.8	*Goniastrea*	11.0 ± 3.8
**Flamenco Beach**	MIS5e	Reef slope	3	7.8 ± 3.4	29.3 ± 0.9	*Porites*	42.9 ± 1.9
**Wadi Nakari**	MIS5e	Reef edge	3	7.6 ± 2.7	31.5 ± 0.5	*Porites*	21.8 ± 1.4
**Wadi Nakari**	MIS5e	Reef slope	2	0.9 ± 0.4	37.8 ± 1.3	*Porites*	33.4 ± 1.0
**Sharm El Luli**	MIS5e	Reef edge	3	0	12.7 ± 2.6	*Porites*	72.9 ± 2.3
**Sharm El Luli**	MIS5e	Reef slope	3	0	37.7 ± 1.5	*Porites*	30.6 ± 0.7
**Sharm El Quibli**	MIS5e	Reef edge	3	0	19.5 ± 1.4	*Porites*	54.3 ± 2.1
